# TCM-CoT-RAG: a chain-of-thought enhanced retrieval-augmented generation system for clinical decision support in Traditional Chinese Medicine rheumatology

**DOI:** 10.3389/fphar.2026.1884526

**Published:** 2026-08-12

**Authors:** Bingbing Fan, Yunlong Fang, Zihan Wang, Fang Ma

**Affiliations:** 1 Department of Rheumatology and Immunology, Xiyuan Hospital, China Academy of Chinese Medical Sciences, Beijing, China; 2 National Center for Integrative Medicine, Department of TCM Rheumatism, China-Japan Friendship Hospital, Beijing, China

**Keywords:** large language models (LLMs), prescription recommendation, retrieval-augmented generation (RAG), syndrome differentiation, traditional Chinese medicine (TCM)

## Abstract

**Background:**

Traditional Chinese Medicine (TCM) rheumatology presents unique challenges for AI-assisted clinical decision support, as the diagnostic process relies heavily on tacit knowledge and individualized reasoning. While Large Language Models (LLMs) have shown promise in medical applications, they remain limited by hallucination risks and inability to replicate expert TCM reasoning. Retrieval-Augmented Generation (RAG) offers a potential solution, yet its application to complex TCM dialectical reasoning remains underexplored.

**Methods:**

We developed TCM-CoT-RAG, a hybrid framework combining RAG with Chain-of-Thought (CoT) prompting, grounded in 1,700 expert-curated clinical cases (1,600 for RAG retrieval; 100 for evaluation, including 50 for blinded expert review by three senior TCM rheumatologists). Deployed on Alibaba Cloud, the five system leverages state-of-the-art LLMs (DeepSeek-V3, Qwen3-235B) under a human-in-the-loop paradigm. We designed a dual-tier evaluation: (1) Objective extraction tasks (Task 1–2) quantified using F1-scores; (2) Generative tasks (Task 3–5) assessed using BERTScore. Two senior TCM rheumatologists (≥15 years clinical experience) blindly assessed model outputs, and a senior chief expert quantified consistency between model predictions and ground truth (GT). Comprehensive ablation studies (S1-S4, S-Skip) isolated the contributions of each CoT module.

**Results:**

TCM-CoT-RAG substantially improved diagnostic accuracy across five LLMs. DeepSeek-V3 with full-chain CoT-RAG achieved Entity F1 of 44.89% (+16.45% over baseline) and Formula F1 of 32.13% (+8.74% over baseline), with BERTScore of 0.81 indicating strong semantic alignment with expert reasoning. Ablation confirmed that the complete CoT pipeline was essential—removing any reasoning module caused performance collapse below the zero-shot baseline. Two independent experts validated clinical utility (Cohen’s κ > 0.7). DeepSeek-V3 achieved the highest ground-truth consistency at 81.6%, and consistency metrics were quantified by the third expert holding the most senior professional title.

**Conclusion:**

This proof-of-concept framework demonstrates the potential of RAG-enhanced CoT reasoning to improve diagnostic consistency in TCM, objectifying the Symptom-Diagnosis-Prescription pipeline. It is important to note that this system is designed as an AI-assisted clinical decision-support tool. All recommendations require validation by qualified TCM practitioners before clinical application.

## 1 Introduction​

**FIGURE 1:**
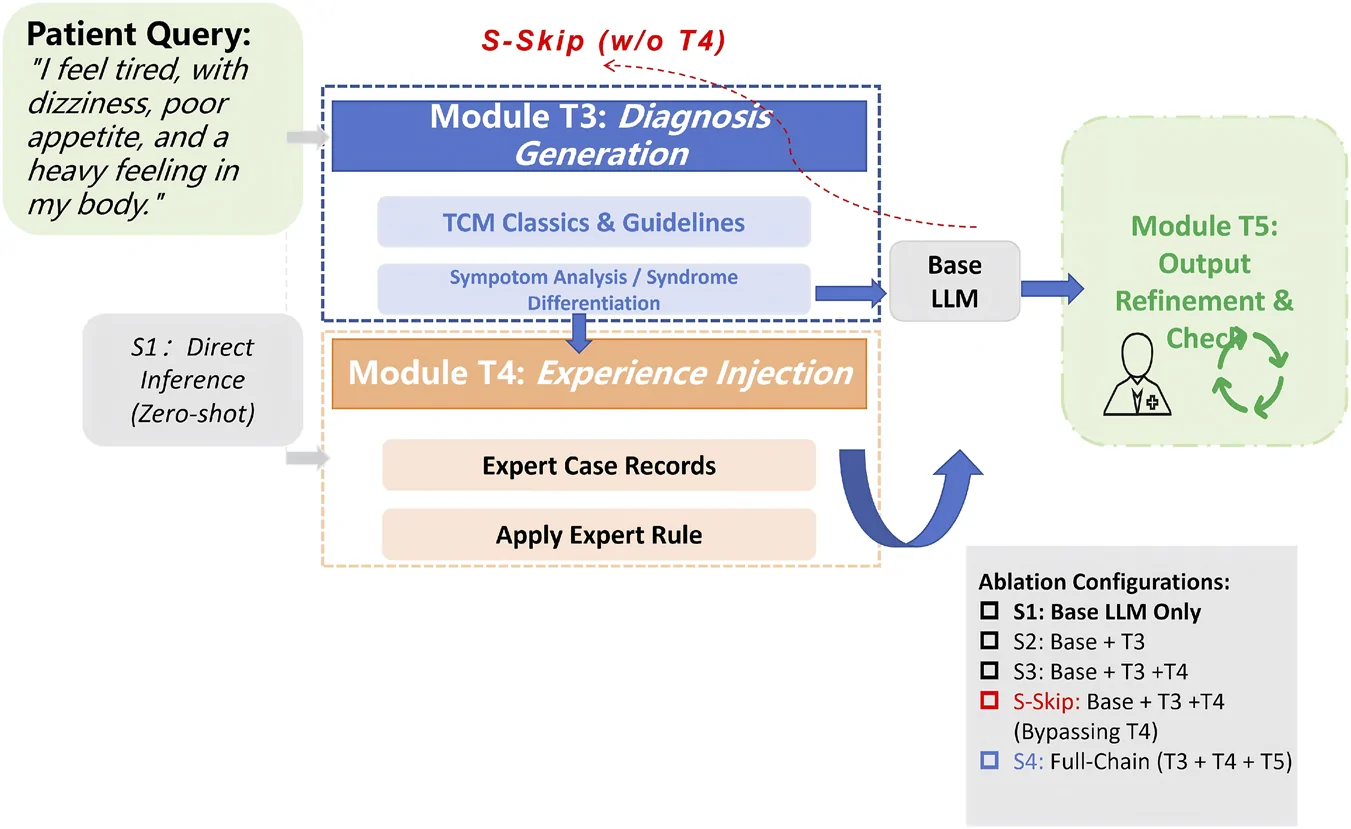
The overview of TCM-CoT-RAG framework with chain-of-thought reasoning and expert critique mechanism.

The digitalization of tacit knowledge remains a critical challenge in specialized fields like Traditional Chinese Medicine (TCM). Bridging static TCM theories with real-world clinical application poses objective difficulties, as pattern differentiation relies heavily on nuanced, individualized judgments. Furthermore, the traditional reliance on individual mentorship introduces profound heterogeneity in how clinical experience is inherited, making the knowledge resistant to standardization (Liu and Gu, 2011). To break away from the confines of conventional “expert mentorship,” a paradigm shift is required. By utilizing structured data extraction and summarization from clinical records (medical cases) as our primary entry point, we aim to construct a standardized workflow in Figure 1. This data-driven approach not only mitigates the subjectivity inherent in traditional mentorship but also provides a scalable framework to digitize and perpetuate TCM expertise (Tang et al., 2024).

The advent of large language models (LLMs) has offered a promising pathway for tackling complex knowledge-intensive tasks, demonstrating remarkable capabilities in natural language understanding and generation. In the medical domain, LLMs hold the potential to revolutionize clinical decision support, patient education, and knowledge management (Lan et al., 2020; Griffiths et al., 2010). However, their application in safety-critical fields like medicine is fraught with peril. A primary concern is the phenomenon of hallucination, where models generate plausible-sounding but factually incorrect or unsupported information, a problem shown to be prevalent even in advanced models like GPT-4 when applied to clinical scenarios (Xie et al., 2010; Chen et al., 2019). Furthermore, LLMs suffer from inherent knowledge limitations: their static training data leads to outdated information (knowledge lag) and an inability to incorporate the latest medical findings (Ton, 2015). Most critically, their responses are often opaque and lack verifiable grounding in authoritative sources, rendering them unreliable for direct clinical application without rigorous safeguards (Xijin et al., 2007; Jung, 2025).

**FIGURE 2:**
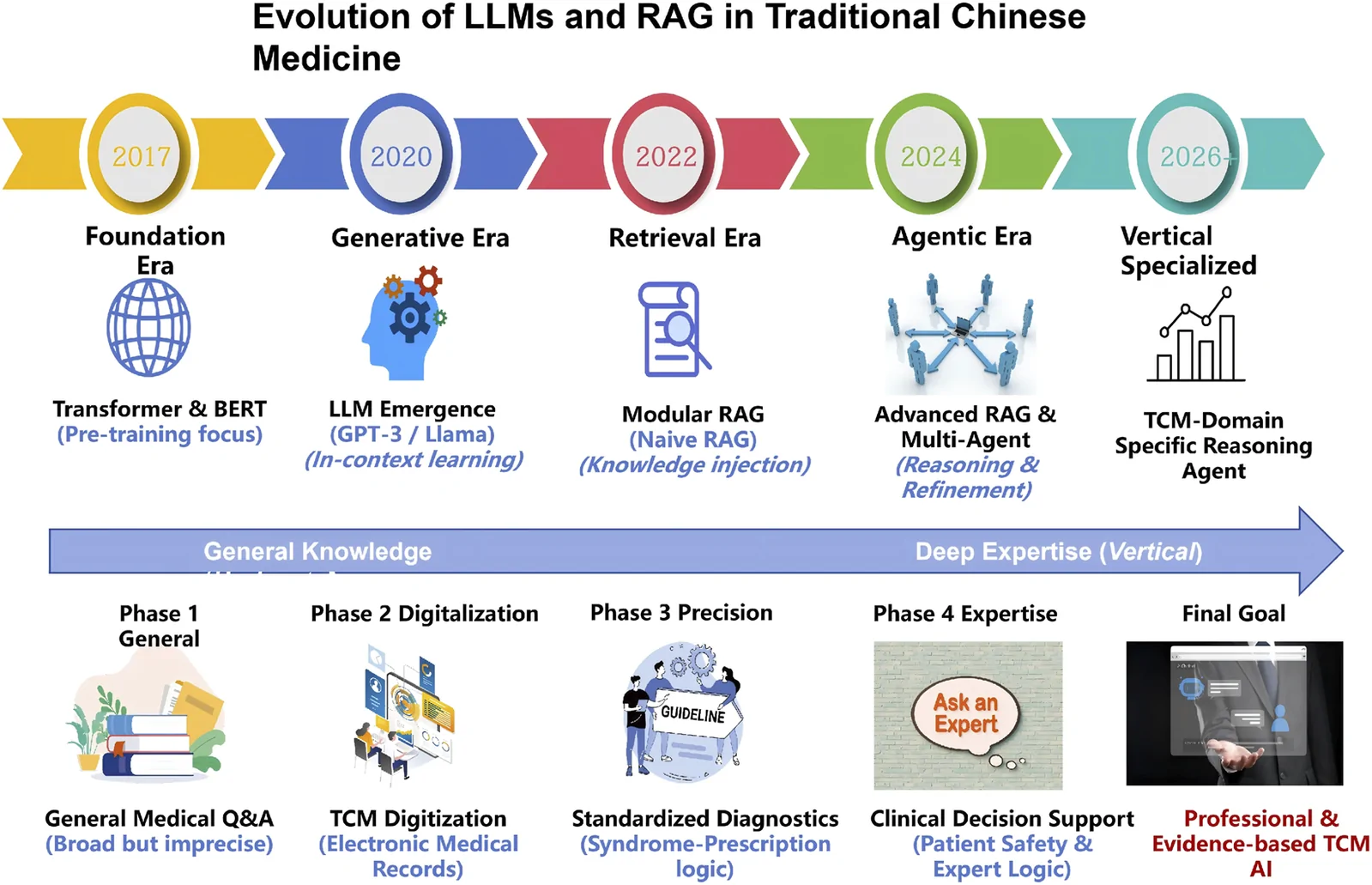
The evolutionary roadmap of LLMs and RAG technology towards specialized TCM applications. The timeline illustrates the progression from general-purpose LLMs to domain-adapted models. The emergence of RAG (2020) addressed the hallucination issue. Early medical LLMs focused on general knowledge. The current stage necessitates Vertical Specialization to address unique challenges such as complex dialectical reasoning, which general TCM models cannot adequately handle.

To mitigate these limitations, Retrieval-Augmented Generation (RAG) has emerged as a powerful and increasingly adopted framework (Lewis et al., 2020). RAG enhances LLMs by integrating an external knowledge retrieval component. Before generating a response, the model first queries a curated database to fetch relevant, up-to-date information and uses this evidence to inform and ground its output. It significantly reduces hallucinations by constraining generation to retrieved contexts (Thesen and Park, 2025; Hanmante et al., 2025), addresses knowledge latency by allowing the underlying database to be updated independently of the model, and enhances transparency by providing citable references for generated content. Recent advancements in Advanced RAGand Self-RAGframeworks have further improved retrieval quality and answer verifiability, demonstrating strong performance in specialized domains, making RAG a compelling approach for building reliable and trustworthy AI systems in medicine in Figure 2.

## 2 The paradigm shift from static LLMs to RAG in healthcare

Large Language Models (LLMs) have demonstrated profound potential in clinical decision support, demonstrating strong capabilities in comprehending complex medical narratives (Clusmann et al., 2023). However, their inherent reliance on static, pre-trained parameters inevitably leads to hallucinations, generating plausible but medically unverified outputs, which remains the most critical barrier to their deployment in safety-critical clinical environments (Wornow et al., 2023). To overcome this, Retrieval-Augmented Generation (RAG) has emerged as an indispensable architecture in medical AI (Zakka et al., 2024). By dynamically anchoring generative outputs to external, rigorously verified knowledge bases, RAG bypasses the limitations of knowledge lag and ensures that clinical advice is factually traceable and medically substantiated, establishing a new baseline for AI reliability in healthcare.

Traditional Chinese Medicine (TCM), characterized by its holistic philosophy and archaic terminology, presents unique hurdles for general-purpose LLMs. Consequently, recent efforts have focused on domain-specific adaptations, evolving through three distinct phases. Initial models like TCMChat (Dai et al., 2024) utilized instruction-tuning to improve conversational fluency regarding TCM concepts (Dai et al., 2024). Subsequently, foundational models such as BianCang (Wei et al., 2025) attempted to internalize broad structured and unstructured data to support general syndrome differentiation (Bian Zheng) (Wei et al., 2025). Most recently, sub-specialized assistants like GastroTCM (Wang et al., 2026) have proven the efficacy of narrow-domain fine-tuning for specific diseases (Wang et al., 2026). Despite these advancements, a fundamental bottleneck persists. These models primarily embed knowledge statically within their parameters, leaving them susceptible to hallucinations. More critically, while they successfully master “textbook-level” vocabulary, they lack the capacity to replicate the nuanced, experience-based reasoning logic of elite physicians.

The quintessential diagnostic process in TCM, “Syndrome Differentiation and Treatment” (Bian Zheng Lun Zhi), is not a simple mapping of symptoms to, but a complex, multi-step cognitive chain. This tacit knowledge is best preserved in the authentic clinical case records of master practitioners. While standard RAG approaches excel at retrieving relevant text snippets, they often fall short in complex clinical reasoning tasks that require step-by-step diagnostic logic (Kanjee et al., 2023). TCM inherently relies on holistic and highly interconnected reasoning networks, traditionally modeled by Knowledge Graphs (KGs) (Long et al., 2024; Qu et al., 2024). However, constructing exhaustive TCM KGs is labor-intensive and often struggles to capture the unstructured nuances of clinical case narratives. While recent advancements such as GraphRAG demonstrate the potential of unifying LLMs with semantic topologies (Pan et al., 2024; Edge et al., 2024), our standard vector-based RAG framework proves highly effective in dynamically retrieving vast, unstructured classic texts, serving as a flexible and scalable alternative to rigid graph structures.

## 3 Methodology

### 3.1 Construction of the three-layer integrated knowledge base and foundational model

**FIGURE 3:**
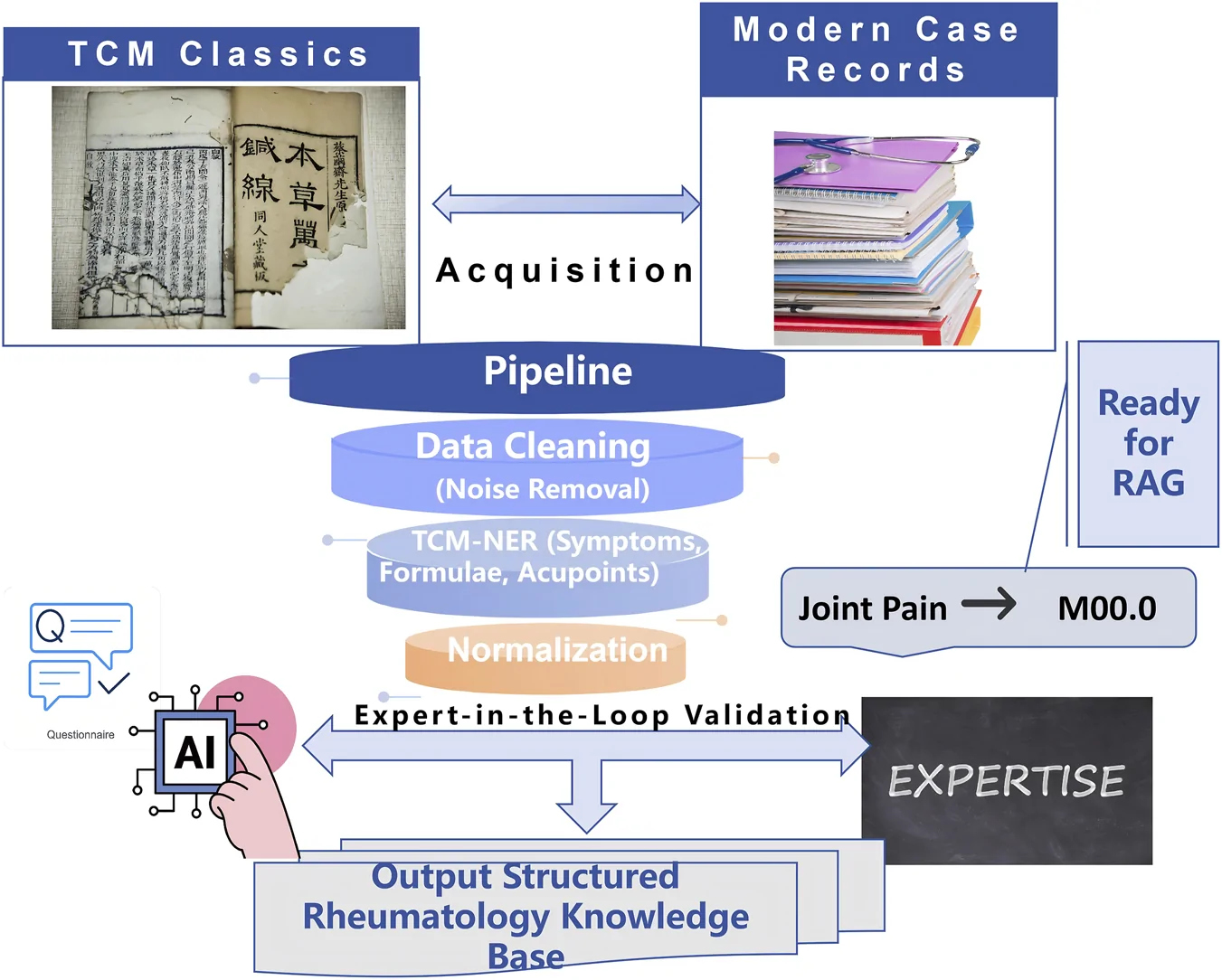
The pipeline for constructing the High-Quality TCM Rheumatology Dataset.

This study employs a multi-stage approach to construct a dataset for Traditional Chinese Medicine (TCM) syndrome differentiation analysis, ensuring data quality and reliability in Figure 3.

Layer 1: Classical Canonical Knowledge: Digital texts of 843 authoritative ancient TCM texts, including Huangdi Neijing, Shanghan Lun, Jingui Yaolue, Bencao Sibian Lu, and Shanghan Mingli Lun, were obtained from verified digital repositories.

Layer 2: Modern Clinical Guidelines: Thirty-five (35) contemporary clinical guidelines and expert consensus documents on rheumatologic diseases (both TCM and integrative medicine) were collected from professional society publications and academic databases.

Layer 3: Expert Experience Archives: A specialized corpus of medical records from renowned master TCM physicians, with a focus on Professor Fang’s case studies, was compiled. This corpus was enriched with in-depth annotations and commentaries provided by senior TCM rheumatologists holding advanced professional titles, capturing nuanced diagnostic reasoning and treatment modifications.

### 3.2 Retrieval hyperparameters and text chunking strategy

Each knowledge layer underwent specialized processing to facilitate machine comprehension and retrieval. The expert case repository (Layer 3) and classical texts corpus were processed using a dense retrieval-oriented vectorization pipeline. Specifically, we first performed semantic chunking using RecursiveCharacterTextSplitter (chunk size: 1,000 characters, overlap: 200 characters) to preserve contextual continuity. All text passages were then converted into dense vector embeddings (1,536 dimensions) using the text-embedding-v4 model via Alibaba Cloud DashScope API. At query time, our retrieval system employs similarity search with Chroma vector database, returning the top-k most semantically relevant chunks based on cosine similarity scores.

To ensure reproducibility, we provide a comprehensive specification of our retrieval system. Top-k retrieval parameters were set to k = 3 for the expert case repository and clinical guidelines, and k = 2 for classical texts (due to higher semantic redundancy). The specific configuration is summarized in Supplementary Appendix. We adopted a RecursiveCharacterTextSplitter with overlapping chunks. Our system employs dense retrieval with cross-encoder re-ranking. The “hybrid search” refers to the combination of BGE-M3 dense vector retrieval and BGE-Reranker for re-ranking. This approach was selected because dense retrieval excels at capturing semantic similarity in TCM terminology, where the same concept may be expressed using diverse classical and modern phrasing. All prompt templates follow a structured format: role specification → task decomposition → output constraints → clinical safety reminders. Complete templates for each reasoning stage (T1–T5) are provided in Supplementary Appendix.

### 3.3 Mechanism of action: explainable chain-of-thought (CoT)

The generation process is structured into a four-stage causal dependency chain: Syndrome Differentiation (Dialectical Analysis): The model first analyzes clinical symptoms to identify the pathological pattern (Bian Zheng). Tacit Knowledge Externalization (Clinical Insight): Conditioning on the analysis above, the model articulates the practitioner’s unique “personal insights” and “experience,” effectively making implicit clinical wisdom explicit. Simulated Expert Critique (Mentor Review): A critical review module simulates a senior mentor’s perspective to valid and refine the reasoning logic. Final Clinical Decision (Diagnosis & Prescription): Utilizing the cumulative context from the previous three stages (long-term context dependency), the model generates the final disease diagnosis and prescription (Fang Ji).

**FIGURE 4:**
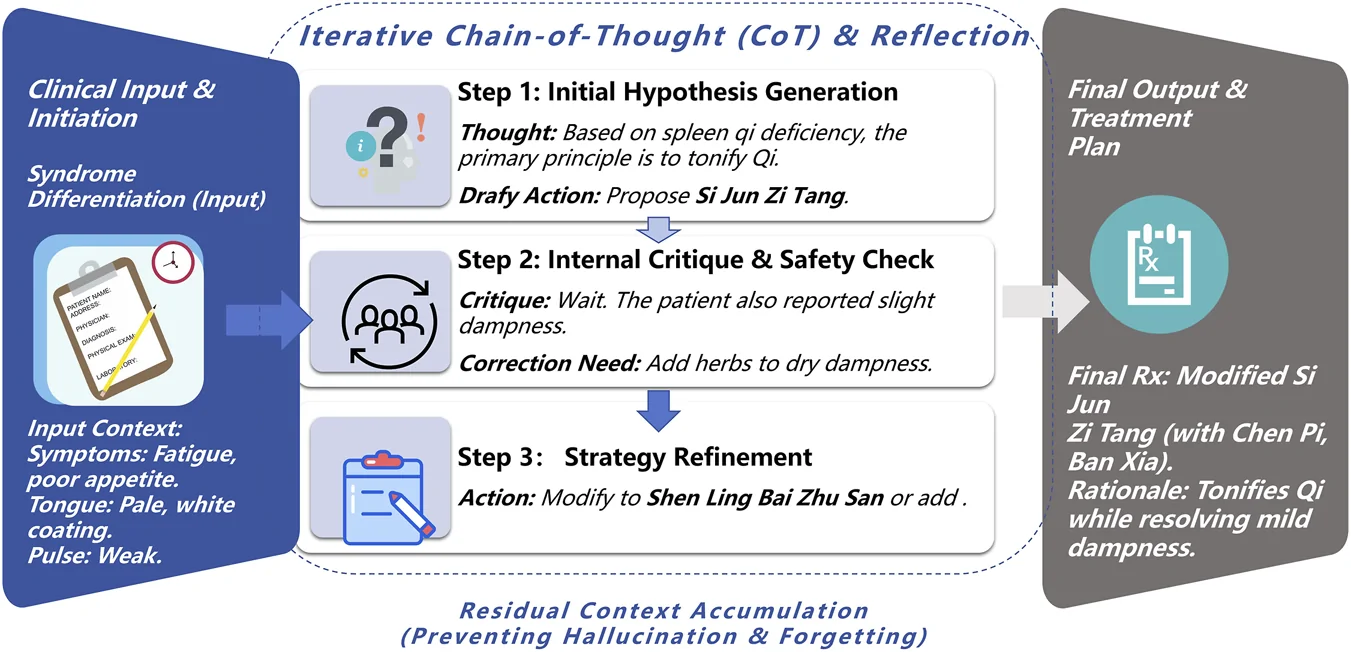
Emulating the TCM expert cognitive process via a retrieval-augmented reasoning chain.

This architecture implements a Chain-of-Thought (CoT) reasoning strategy, which decomposes complex clinical decision-making into intermediate logical steps. By enforcing the generation of “Insights” and “Critiques” prior to the final prescription, we achieve two key scientific objectives. The logical flow ensures that the prescription (Fang) strictly follows the therapeutic method (Fa) and pathology (Li), preventing the model from hallucinating treatments disconnected from the diagnosis. The intermediate outputs serve as a transparent “reasoning trajectory,” allowing clinicians to verify why a prescription was made, rather than just what was prescribed. By embedding the “Insights” module, the model effectively learns the stylistic and cognitive patterns of the specific famous practitioner, capturing not just medical data but the “art” of their practice as shown in Figure 4.

### 3.4 Evaluation metrics

We designed a dual-tier evaluation protocol: For objective extraction tasks (Task 1: Case Key-point Prediction; Task 2: Formula Prediction), performance was quantified using F1-scores. For complex generative tasks (Task 3: Syndrome Differentiation; Task 4: Experience Summary; Task 5: Supervisor Evaluation), we employed BERTScore to quantitatively assess the semantic fidelity and logical alignment of the LLMs’ diagnostic reasoning against authoritative clinical guidelines. Given that this study evaluates output quality against ground truth annotations on a fixed evaluation set (rather than training a model), traditional cross-validation was not applicable. Instead, we employed a comprehensive evaluation strategy consisting of: (1) automated metric evaluation on the full 100-case evaluation set, (2) stratified sampling of 50 cases for blinded expert assessment by three independent senior TCM rheumatologists, and (3) ablation studies to validate the contribution of each framework component.

#### 3.4.1 Objective extraction metrics (task 1 & task 2)

For entity-level prediction tasks, Case Key-point Prediction (Task 1) and Formula Prediction (Task 2), we employed F1-score as the primary evaluation metric. F1-score is the harmonic mean of precision and recall, providing a robust assessment even when class distributions are imbalanced, which is characteristic of TCM clinical datasets where certain syndrome patterns or formula compositions may be underrepresented.

#### 3.4.2 Semantic fidelity metrics (task 3, 4 & 5)

For complex generative tasks requiring clinical reasoning assessment, Syndrome Differentiation (Task 3), Experience Summary (Task 4), and Supervisor Evaluation (Task 5). We employed BERTScore to quantify the semantic similarity between model-generated outputs and expert-provided gold standards.

#### 3.4.3 Ablation study design

To isolate the individual contributions of each module within our Chain-of-Thought reasoning pipeline, we conducted comprehensive ablation studies by progressively removing or bypassing individual components. Performance degradation at intermediate stages (S2, S3) and recovery upon full-chain integration (S4) empirically validate the necessity of the complete closed-loop reasoning pathway for TCM clinical decision-making.

#### 3.4.4 Expert clinical evaluation (qualitative assessment)

Beyond automated metrics, we conducted qualitative expert evaluation to assess clinical applicability and safety. Two senior TCM rheumatologists (each with >15 years of clinical experience) independently reviewed a random sample of 5 model-generated outputs across 250 tasks. Another senior professional title TCM rheumatology specialists performed consistency scoring independently. Experts rated outputs on a 5-point Likert scale for: Clinical reasoning coherence, Diagnostic accuracy alignment with TCM theory, Prescription appropriateness, Overall clinical utility as a decision support tool. The inter-rater reliability was assessed using Cohen’s Kappa (κ), with κ > 0.7 indicating substantial agreement among evaluators.

### 3.5 Ethics statement and large language models selection

This study was conducted in accordance with the Declaration of Helsinki. The use of de-identified patient clinical records for this retrospective analysis was approved by the Ethics Committee of Xiyuan Hospital, China Academy of Chinese Medical Sciences (Approval No.2025XLA088).

In this study, we selected five representative LLMs: DeepSeek, Qwen, GPT, Kimi, and GLM (Zheng et al., 2023). The five base models (DeepSeek-V3, Qwen3-235B-A22B, GPT-5-Nano, Kimi, and GLM-4.6) were accessed via their respective commercial APIs with their pre-trained, frozen weights, with the temperature parameter consistently set to 0.01. Domain adaptation was achieved exclusively through the RAG retrieval mechanism and the CoT prompting framework. Detailed specifications of these models are presented in Table 1. The interactive web inference platform of our TCM-CoT-RAG system is deployed at http://47.239.72.25:8501. This platform requires a login password to protect confidential clinical case data; researchers who wish to replicate our experiments may contact the corresponding author to obtain access credentials.

**TABLE 1:** Detailed information of the large language models included in this study.

Model name	Developer	Parameters	Details	Release date
DeepSeek (Liu et al., 2024)	DeepSeek AI	671B	DeepSeek-V3	20 November 2025
Qwen (Yang et al., 2025)	Alibaba Cloud	235B	Qwen3-235B-A22B	15 December 2025
GPT (Singh et al., 2025)	OpenAI	8B	GPT-5-Nano	5 January 2026
Kimi (Xu et al., 2023)	Moonshot AI	20B	Moonshot-Kimi-K2-Instruct	10 October 2025
GLM (Glm et al., 2024)	Zhipu AI	32B	GLM-4.6	1 December 2025

### 3.6 Statistical analysis

All experiments were conducted using deterministic inference (temperature = 0.01) across three independent runs on the fixed 100-case evaluation set. Results are reported as mean ± standard deviation (SD) across three runs. For Entity F1 and Formula F1, 95% confidence intervals were derived using the Wilson score interval method (appropriate for proportion-based metrics computed on the 100-case evaluation set). For BERTScore, within-run case-level mean ± SD is reported, and the run-level SD across three runs is provided to demonstrate result stability. Statistical significance of model comparisons was assessed using Friedman test for overall multi-group comparison and Mann-Whitney U with Bonferroni correction (α = 0.005) for *post hoc* pairwise comparisons. Effect sizes were quantified using Cohen’s d.

## 4 Results​

### 4.1 Dataset and knowledge base

To enable rigorous evaluation of our framework’s generalization capability while preventing data leakage, we partitioned the dataset into two completely independent patient populations (The structure of the database is as shown in Table 2). The 1,700 expert clinical cases were partitioned as follows: (1) RAG Knowledge Base: 1,600 cases (94.1%) — for retrieval augmentation; (2) Evaluation Set: 100 cases (5.9%) — for automated model evaluation; (3) Of the 100 evaluation cases, 50 were selected via stratified sampling for blinded expert assessment by three senior TCM rheumatologists. A fundamental principle in our partitioning strategy was strict patient-level separation between the RAG Knowledge Base and the Evaluation Set—no patient appears in both partitions. This separation ensures that during evaluation, the models cannot retrieve cases from the same patient that they are being tested on, thereby assessing true out-of-distribution generalization rather than memorization.

**TABLE 2:** Schema and attributes of the standardized TCM medical case dataset.

Field name	Description
Original Text​	The master’s original, unaltered notes for the case, preserving the authentic language and thought process used during the consultation
Date of Record​	The date when the case was documented, allowing for chronological tracking and seasonal analysis of symptoms and treatments
Patient Gender​	The gender of the patient, a fundamental factor considered in TCM diagnosis and treatment planning to address physiological differences
Patient Age​	The age of the patient, crucial for understanding constitutional strengths and weaknesses according to TCM life stage theories
Symptoms​	A detailed description of the patient’s chief complaints and accompanying symptoms, forming the basis for TCM pattern differentiation (Bian Zheng)
Case Summary​	The master’s concise summary of the case’s core elements, including the identified TCM pattern (e.g., Liver Qi Stagnation, Spleen Qi Deficiency) and the key treatment strategy
Recommendations & Prescriptions​	The master’s prescribed treatment plan, which may include (with specific ingredients and dosages), acupuncture point selections, dietary advice, and lifestyle recommendations

To characterize the representativeness of our single-expert knowledge base and contextualize the scope of the evaluation, we analyzed the distribution of rheumatic diseases and TCM syndrome patterns. The 1,700 cases span eight major rheumatic disease categories in Table 3.

**TABLE 3:** Distribution of disease types and traditional chinese medicine syndrome patterns in included cases.

Disease type	Number of cases	TCM syndrome pattern	Number of cases
Arthritis (general)	337	Wind-Dampness Blockage Syndrome	213
Rheumatoid Arthritis	254	Damp-Heat Internal Accumulation Syndrome	117
Ankylosing Spondylitis	126	Qi-Yin Deficiency Syndrome	72
Sjögren’s Syndrome	107	Blood Stasis Blocking Collaterals Syndrome	70
Osteoporosis	66	Meridian Blood Stasis Blockage Syndrome	68
Systemic Lupus Erythematosus	55	Qi Stagnation and Blood Stasis Syndrome	62
Gouty Arthritis	48	Qi Deficiency and Blood Stasis Syndrome	62
Takayasu Arteritis	23	Damp-Heat Accumulation Syndrome	60

### 4.2 End-to-end performance evaluation

#### 4.2.1 Experimental setup and evaluation tasks

To systematically evaluate the contribution of each reasoning component within the TCM-CoT-RAG framework, we conducted a comprehensive ablation study across five large language models from diverse developers. We designed experimental configurations to isolate the contribution of each module. Task 1: Information Extraction, Structured extraction of clinical entities (symptoms, diagnosis, treatment principles) from patient records. Task 2: Formula Prediction, Generation of botanical drug prescriptions based on syndrome differentiation. Given that Tasks 1 and 2 represent the ultimate clinical workflow outputs, we selected F1-score as the primary evaluation metric for the ablation study. The intermediate generation tasks (Tasks 3, 4, and 5) serve as implicit Chain-of-Thought rationales. Their impact is best reflected by observing how their presence or absence affects the final objective prediction. The ablation results demonstrate that the complete TCM-CoT-RAG pipeline (S4: Full-Chain) substantially outperforms both the zero-shot baseline and all intermediate configurations across all evaluated models.

Across all evaluated models, the complete proposed pipeline (S4: Full-Chain, integrating T3 Diagnosis, T4 Experience, and T5 Formulation) substantially outperformed both the zero-shot baseline (S1) and all intermediate configurations in Table 4. For instance, utilizing the DeepSeek-v3 model, the S4 configuration achieved the highest overall performance, reaching an Entity F1 of 44.89% ± 0.53% (95% CI: 43.55%–46.23%, an absolute increase of +16.45% over the baseline) and a Formula F1 of 32.13% ± 0.42% (95% CI: 31.10%–33.16%, Δ +8.74% vs. baseline). Similar substantial improvements were observed in Qwen3-235b-a22b (+17.47% in Entity F1; +8.80% in Formula F1) and Kimi (+18.15% in Entity F1; +12.32% in Formula F1). A counter-intuitive yet theoretically profound phenomenon was observed during the intermediate ablation stages. When the models were prompted with only partial steps of the reasoning chain, such as adding only the Diagnosis step (S2: + T3) or Diagnosis plus Experience without the final synthesis (S3: + T3 + T4), performance universally dropped below the raw zero-shot baseline (S1). For example, DeepSeek-v3’s Entity Extraction F1 plummeted by −22.61% under the S2 configuration compared to S1. We attribute this “performance penalty” to contextual fragmentation. The S-Skip configuration (T3 + T5, bypassing the T4 Experience module) was designed to test whether the model could jump directly from Diagnosis to Prescription. Across all models, bypassing the T4 step resulted in severe performance degradation, particularly in the core Formula Prediction task. Most notably, Qwen3’s Formula F1 collapsed to a mere 2.98% (a −11.63% drop from the baseline) under S-Skip, whereas it achieved 23.41% under the S4 Full-Chain.

**TABLE 4:** Ablation results demonstrating the holistic synergy of T3, T4, and T5 modules in TCM clinical reasoning.

Model & Configurations	Information extraction	Performance	Formula prediction	Performance
	ΔΔ over base		ΔΔ over base	
	Mean ± SD, SE, 95%CI	ΔΔ F1 (%)	Mean ± SD, SE	ΔΔ F1 (%)
DeepSeek-V3				
S1: Base (LLM only)	28.44 ± 0.31, 0.18, [27.66,29.22]	​	23.39 ± 0.37, 0.21, [22.49,24.29]	​
S2: + T3 (Diagnosis)	5.83 ± 0.21, 0.12, [5.31,6.35]	−22.61%	15.59 ± 0.30, 0.17, [14.86,16.32]	−7.80%
S3: + T3 + T4 (Experience)	9.80 ± 0.29, 0.17, [9.07,10.53]	−18.64%	12.84 ± 0.32, 0.18, [12.06,13.62]	−10.55%
S-Skip: + T3 + T5 (w/o T4)	5.44 ± 0.23, 0.13, [4.88,6.00]	−23.00%	13.99 ± 0.32, 0.18, [13.21,14.77]	−9.40%
S4: Full-Chain (T3+T4+T5)	44.89 ± 0.53, 0.31, [43.55,46.23]	16.45%	32.13 ± 0.42, 0.24, [31.10,33.16]	8.74%
Qwen3-235b-a22b				
S1: Base (LLM only)	22.41 ± 0.30, 0.17, [21.68,23.14]	​	14.61 ± 0.32, 0.18, [13.83,15.39]	​
S2: + T3 (Diagnosis)	6.40 ± 0.29, 0.17, [5.67,7.13]	−16.01%	15.51 ± 0.32, 0.18, [14.73,16.29]	0.90%
S3: + T3 + T4 (Experience)	5.67 ± 0.27, 0.15, [5.02,6.32]	−16.74%	15.93 ± 0.33, 0.19, [15.11,16.75]	1.32%
S-Skip: + T3 + T5 (w/o T4)	4.46 ± 0.25, 0.14, [3.86,5.06]	−17.95%	2.98 ± 0.26, 0.15, [2.34,3.62]	−11.63%
S4: Full-Chain (T3+T4+T5)	39.87 ± 0.44, 0.25, [38.79,40.95]	17.47%	23.41 ± 0.35, 0.20, [22.55,24.27]	8.80%
gpt-5-nano				
S1: Base (LLM only)	24.19 ± 0.32, 0.18, [23.41,24.97]	​	1.27 ± 0.26, 0.15, [0.63,1.91]	​
S2: + T3 (Diagnosis)	10.72 ± 0.35, 0.20, [9.86,11.58]	−13.47%	3.47 ± 0.29, 0.17, [2.74,4.20]	2.20%
S3: + T3 + T4 (Experience)	6.23 ± 0.29, 0.17, [5.50,6.96]	−17.96%	1.69 ± 0.29, 0.17, [0.96,2.42]	0.42%
S-Skip: + T3 + T5 (w/o T4)	5.96 ± 0.29, 0.17, [5.23,6.69]	−18.23%	1.44 ± 0.29, 0.17, [0.71,2.17]	0.17%
S4: Full-Chain (T3+T4+T5)	30.51 ± 0.36, 0.21, [29.61,31.41]	6.32%	9.89 ± 0.34, 0.20, [9.03,10.75]	8.62%
glm-4.6				
S1: Base (LLM only)	15.45 ± 0.31, 0.18, [14.67,16.23]	​	11.31 ± 0.33, 0.19, [10.49,12.13]	​
S2: + T3 (Diagnosis)	6.87 ± 0.30, 0.17, [6.14,7.60]	−8.58%	8.00 ± 0.33, 0.19, [7.18,8.82]	−3.31%
S3: + T3 + T4 (Experience)	6.60 ± 0.30, 0.17, [5.87,7.33]	−8.85%	1.21 ± 0.29, 0.17, [0.48,1.94]	−10.10%
S-Skip: + T3 + T5 (w/o T4)	6.59 ± 0.30, 0.17, [5.86,7.32]	−8.86%	1.15 ± 0.29, 0.17, [0.42,1.88]	−10.16%
S4: Full-Chain (T3+T4+T5)	31.23 ± 0.34, 0.20, [30.37,32.09]	15.78%	14.87 ± 0.34, 0.20, [14.01,15.73]	3.56%
Moonshot-Kimi-K2-Instruct				
S1: Base (LLM only)	20.89 ± 0.32, 0.18, [20.11,21.67]	​	9.86 ± 0.34, 0.20, [9.00,10.72]	​
S2: + T3 (Diagnosis)	6.83 ± 0.30, 0.17, [6.10,7.56]	−14.06%	17.07 ± 0.36, 0.21, [16.17,17.97]	7.21%
S3: + T3 + T4 (Experience)	6.91 ± 0.30, 0.17, [6.18,7.64]	−13.98%	16.83 ± 0.37, 0.21, [15.93,17.73]	6.96%
S-Skip: + T3 + T5 (w/o T4)	6.62 ± 0.30, 0.17, [5.89,7.35]	−14.26%	17.31 ± 0.36, 0.21, [16.41,18.21]	7.44%
S4: Full-Chain (T3+T4+T5)	39.04 ± 0.36, 0.21, [38.14,39.94]	18.15%	22.18 ± 0.36, 0.21, [21.28,23.08]	12.32%

S1 represents the baseline model without external enhancement. ΔΔ F1 indicates the absolute performance difference compared to the baseline (S1). S-Skip intentionally omits the experience module (T4) to evaluate logical continuity. The significant performance drop in intermediate stages (S2, S3) and the substantial rebound in the Full-Chain stage (S4) highlight the necessity of a closed-loop reasoning pathway. Entity F1 and Formula F1 are computed on the full 100-case evaluation set; 95% CI, derived via Wilson score interval.

#### 4.2.2 Error analysis: contextual fragmentation phenomenon

Analysis of Intermediate Configuration Performance: We observed that partial reasoning steps (S2, S3) caused performance degradation below the zero-shot baseline (S1). This ‘over-loading effect’ or ‘contextual fragmentation’ occurs because: (1) intermediate reasoning modules without full expert knowledge grounding introduce noisy context that dilutes the model’s attention to critical clinical indicators; (2) the partial modules (T3 syndrome differentiation, T4 experience) are designed to work synergistically with the RAG grounding module (T5). Without T5, T3/T4 produce reasoning trajectories that deviate from the expert’s actual clinical logic. Only the complete pipeline (S4: Full-Chain, T3+T4+T5) achieves synergistic enhancement. To understand the counter-intuitive performance drop in intermediate configurations, we conducted detailed error analysis on representative cases. We attribute the observed “performance penalty” to contextual fragmentation.

In zero-shot setting (S1), LLMs rely on their intrinsic probabilistic associations, which, while superficial, maintain a coherent (albeit shallow) generation pattern. In incomplete pipeline (S2, S3), when forced into a structured but incomplete TCM reasoning chain, models suffer from cognitive overload, they lose their intuitive heuristics without gaining the full benefit of a logical clinical conclusion. The partial modules (T3, T4) are designed to work synergistically with T5: Without T5’s RAG grounding, T3/T4 produce reasoning trajectories that deviate from the expert’s actual clinical logic.

【Error Case 1: S2 Configuration (+T3 only) — DeepSeek-V3】
Chief Complaint: Joint swelling and pain for 12 years, exacerbated for 3 months.
S2 Output (Incorrect):
Syndrome: Kidney Deficiency with Cold-Damp Bi.
Formula: Du Huo Ji Sheng Tang modified.
Reasoning: “Based on symptoms.” [superficial analysis, lacks depth].
Problems: Entity F1: 5.83% (−22.61% vs. baseline) Failed to capture expert’s experiential modifications.
S4 Output (Correct):
Syndrome: Kidney Yang Deficiency, Cold-Damp Encumbrance.
Formula: Du Huo Ji Sheng Tang + Xi Xin 3 g
Reasoning: References “Fang’s Experience Collection”.
Root Cause: T4 module provides essential experiential knowledge.

### 4.3 Reasoning process quality evaluation

The Full-Chain configuration (S4) consistently achieved substantially higher BERTScores across all three reasoning dimensions compared to the zero-shot baseline (S1) in Table 5. For example, DeepSeek-V3 improved from 54.32% → 81.07% (+26.75%) in Syndrome Analysis. Qwen3 improved from 40.97% → 78.06% (+37.09%) in Syndrome Analysis. The largest improvements were observed in Syndrome Analysis, suggesting that the T3 Syndrome Differentiation (Dialectical Analysis) module most effectively captures the expert’s diagnostic reasoning patterns. The retrieval-augmented dialectical grounding helps the model produce reasoning that semantically mirrors expert clinical documentation. GLM-4.6’s near-zero Task 5 baseline reflects its fundamental inability to generate supervisor-style evaluation commentary without domain-specific grounding. The dramatic improvement to 68.61% (+63.80%) under Full-Chain underscores how critical the RAG mechanism is for models with limited inherent TCM knowledge.

**TABLE 5:** Evaluation of diagnostic and reasoning rationales (Base vs. Full-Chain RAG).

Model	Setup	Task 3: syndrome (BERTScore)	Task 4: experience (BERTScore)	Task 5: supervisor (BERTScore)
DeepSeek-V3	S1 (Base)	54.32 ± 0.30, 0.17, [53.58.55.06]	43.09 ± 0.30, 0.17, [42.34,43.84]	48.45 ± 0.34, 0.20, [47.60,49.30]
​	S4 (Full)	81.07 ± 0.37, 0.21, [80.15,81.99]	75.22 ± 0.37, 0.21, [74.31,76.13]	73.54 ± 0.35, 0.20, [72.67,74.41]
qwen3-235b-a22b	S1 (Base)	40.97 ± 0.34, 0.20, [40.12,41.82]	52.05 ± 0.36, 0.21, [51.15,52.95]	50.92 ± 0.36, 0.21, [50.02,51.82]
​	S4 (Full)	78.06 ± 0.37, 0.21, [77.14,78.98]	72.73 ± 0.36, 0.21, [71.83,73.63]	71.97 ± 0.36, 0.21, [71.07,72.87]
gpt-5-nano	S1 (Base)	52.27 ± 0.36, 0.21, [51.37,53.17]	50.51 ± 0.37, 0.21, [49.59,51.43]	53.65 ± 0.36, 0.21, [52.75,54.55]
​	S4 (Full)	69.55 ± 0.36, 0.21, [68.65,70.45]	66.40 ± 0.37, 0.21, [65.48,67.32]	66.57 ± 0.36, 0.21, [65.67,67.47]
Moonshot-Kimi-K2-Instruct	S1 (Base)	52.70 ± 0.36, 0.21, [51.80,53.60]	54.54 ± 0.37, 0.21, [53.62,55.46]	51.78 ± 0.36, 0.21, [50.88,52.68]
​	S4 (Full)	76.64 ± 0.37, 0.21, [75.73,77.54]	69.14 ± 0.37, 0.21, [68.22,70.06]	68.22 ± 0.36, 0.21, [67.32,69.12]
glm-4.6	S1 (Base)	46.07 ± 0.36, 0.21, [45.17,46.97]	41.03 ± 0.37, 0.21, [40.11,41.95]	4.81 ± 0.36, 0.21, [3.91,5.71]
​	S4 (Full)	74.72 ± 0.37, 0.21, [73.80,75.64]	69.79 ± 0.37, 0.21, [68.87,70.71]	68.61 ± 0.36, 0.21, [67.71,69.51]

GLM-4.6’s Task 5 baseline of 4.81% represents an extreme outlier reflecting the model’s poor zero-shot performance on supervisor evaluation, which is discussed in Section 4.2.2.

**FIGURE 5:**
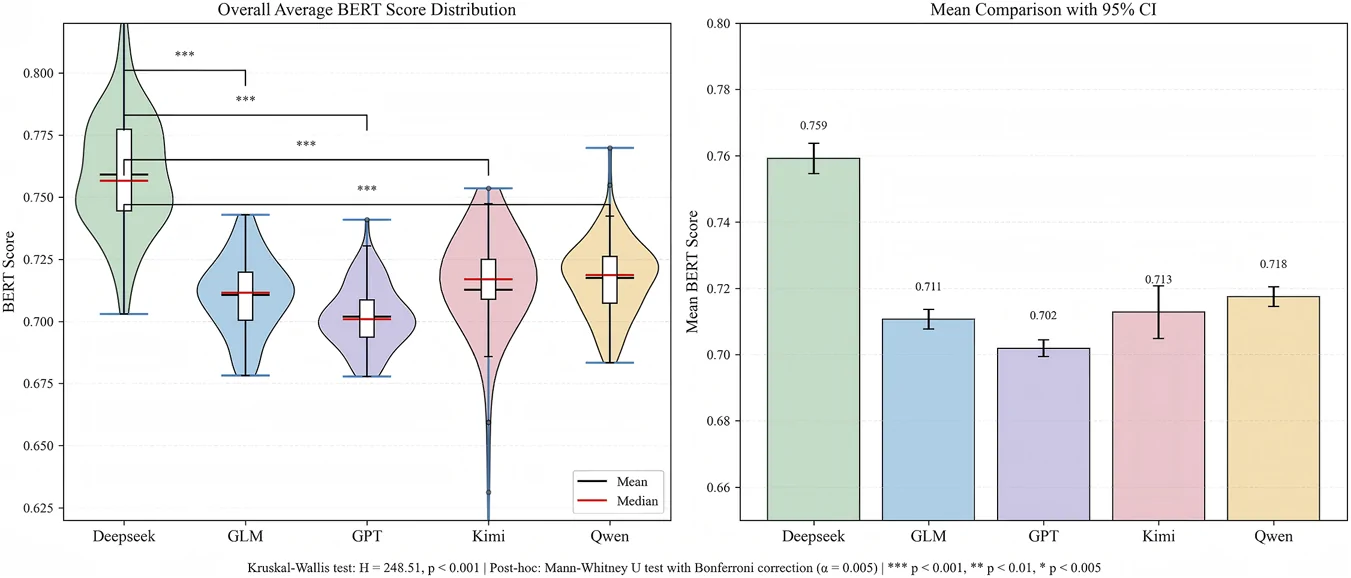
Comparative performance of five large language models across multi-dimensional TCM clinical reasoning tasks.

As shown in Figure 5, DeepSeek-V3 achieved the highest overall BERT score (0.759 ± 0.023, 95% CI [0.755, 0.764]), significantly outperforming all other models (Kruskal–Wallis H = 248.51, p < 0.001; *post hoc* Mann-Whitney U with Bonferroni correction). GLM (0.711 ± 0.015), GPT (0.702 ± 0.013), and Qwen (0.718 ± 0.015) showed comparable performance (all pairwise comparisons: p > 0.005), while Kimi exhibited greater variability (SD = 0.041) with a wider distribution range. DeepSeek-V3 significantly outperformed all other models with large effect sizes (Cohen’s d = 1.40–3.04, all p < 0.001). Among the remaining models, GPT vs. Qwen showed a large effect size (d = −1.11, p < 0.001), while GLM, GPT, Kimi, and Qwen largely exhibited negligible to small pairwise differences (|d| < 0.5, p > 0.005), except for GLM vs. GPT (d = 0.63, medium effect) in Table 6.

**TABLE 6:** Post-hoc pairwise comparisons (mann-whitney U with bonferroni correction).

Comparison	Mean diff	Cohen’s d	Effect size
DeepSeek vs. GLM	0.0485	2.4652	large
DeepSeek vs. GPT	0.0573	3.0432	large
DeepSeek vs. Kimi	0.0464	1.3989	large
DeepSeek vs. Qwen	0.0417	2.1152	large
GLM vs. GPT	0.0088	0.6264	medium
GLM vs. Kimi	−0.0021	−0.0689	negligible
GLM vs. Qwen	−0.0068	−0.4496	small
GPT vs. Kimi	−0.0109	−0.3617	small
GPT vs. Qwen	−0.0156	−1.1099	large
Kimi vs. Qwen	−0.0047	−0.1535	negligible

Bonferroni-corrected α = 0.005 (10 comparisons). Effect size: |d| ≥ 0.8 = large, 0.5 ≤ |d| < 0.8 = medium, 0.2.

### 4.4 Expert human validation

To provide a comprehensive assessment of model performance beyond automated metrics, we conducted expert human validation on Tasks 1 and 2. Due to the intensive nature of blinded expert review, 50 cases were selected from the evaluation set via stratified sampling. Two senior TCM rheumatologists (each with >15 years of clinical experience) independently evaluated the model-generated outputs in a blinded manner. The third expert with the highest professional title quantified the consistency between model predictions and ground truth standard answers.

For Task 1, the expert ratings showed no significant difference among models (Expert 1: H = 8.99, p = 0.061; Expert 2: H = 6.92, p = 0.140), with all models receiving comparable quality scores (range: 3.28–3.78). However, when evaluating consistency with ground truth standard answers, significant differences emerged (H = 17.24, p = 0.0017). DeepSeek-V3 significantly outperformed all other models in ground truth consistency, achieving a mean score of 1.00 ± 0.65 with an 81.6% match rate, substantially higher than GLM (0.64 ± 0.53, 62.0%), GPT (0.60 ± 0.57, 56.0%), Kimi (0.66 ± 0.72, 54.0%), and Qwen (0.52 ± 0.68, 44.0%) (Table 7). Post-hoc pairwise comparisons with Bonferroni correction revealed that DeepSeek-V3’s superiority was significant compared to all other models (all p < 0.01, Cohen’s d = 0.50–0.73, medium effect sizes).

**TABLE 7:** Expert evaluation results for tasks 1 and two.

Model	Task 1 Expert rating	Task 1< GT consistency	Task 1 Match rate	Task 2 Expert rating	Task 2 GT consistency	Task 2 Match rate
DeepSeek	3.78 ± 1.19	1.00 ± 0.65	81.6%	2.83 ± 0.84	2.76 ± 2.32	79.6%
GLM	3.40 ± 1.06	0.64 ± 0.53	62.0%	3.04 ± 0.86	2.82 ± 2.26	88.0%
GPT	3.28 ± 1.11	0.60 ± 0.57	56.0%	2.30 ± 0.61	0.06 ± 0.24	6%
Kimi	3.62 ± 1.01	0.66 ± 0.72	54.0%	2.40 ± 0.70	1.62 ± 2.30	60.0%
Qwen	3.50 ± 1.04	0.52 ± 0.68	44.0%	2.72 ± 0.78	3.26 ± 3.09	74.0%
Kruskal–Wallis p	0.061 (ns)	0.0017**	—	<0.001***	<0.001***	—

Values shown as mean ± SD. GT, Ground Truth. Match Rate = percentage of cases with GT > 0. ***p < 0.001, **p < 0.01, ns = not significant.

**FIGURE 6:**
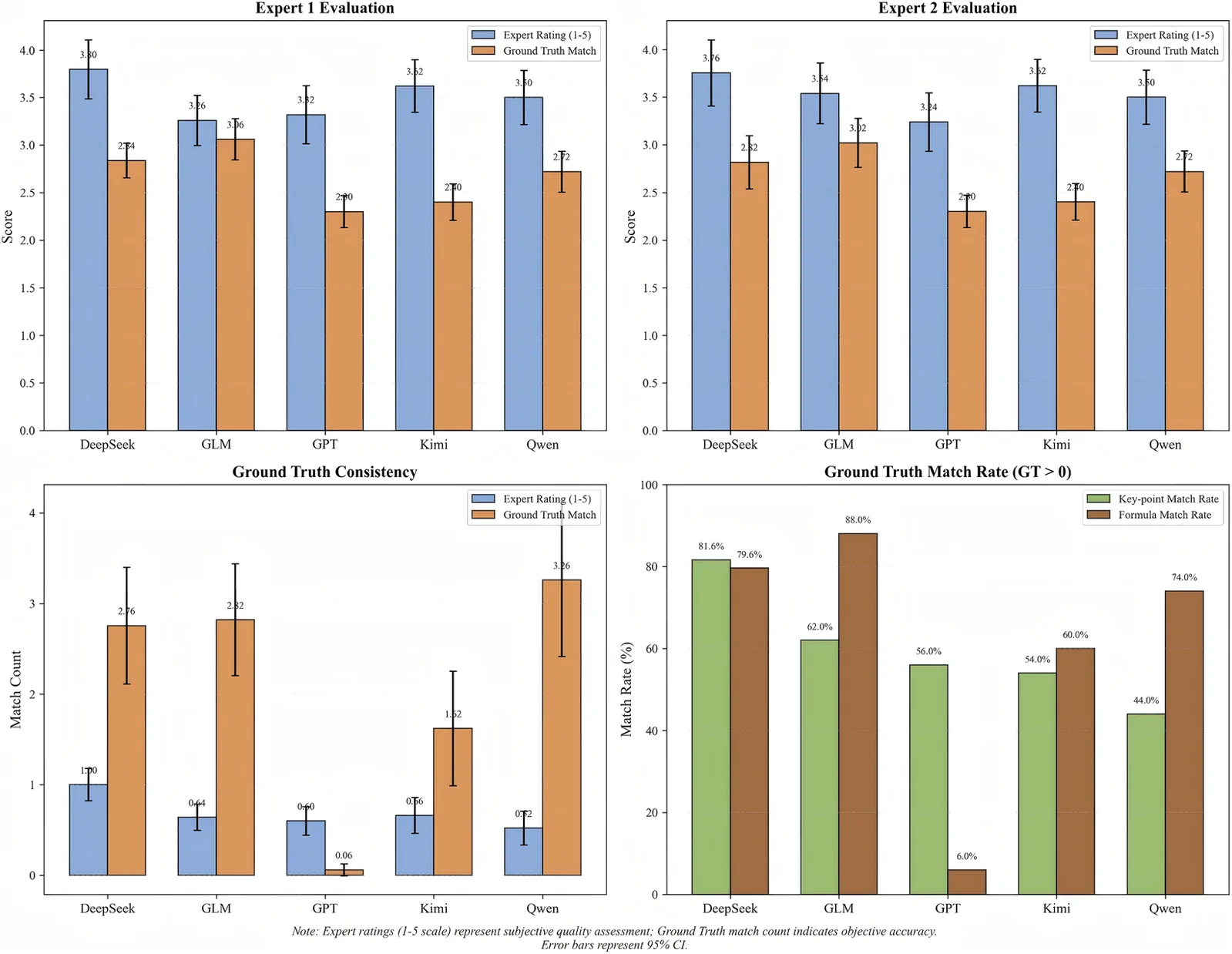
Blinded expert evaluation results:TCM-CoT-RAG (full-chain).

For Task 2, both expert ratings and ground truth consistency showed highly significant differences among models (all Kruskal–Wallis tests: p < 0.001). Notably, GPT demonstrated dramatically poor performance in ground truth consistency, achieving only 0.06 ± 0.24 points with a mere 6.0% match rate, compared to GLM (2.82 ± 2.26, 88.0%), DeepSeek-V3 (2.76 ± 2.32, 79.6%), Qwen (3.26 ± 3.09, 74.0%), and Kimi (1.62 ± 2.30, 60.0%) (Table 7). The expert quality ratings were generally aligned with ground truth consistency, with GLM receiving the highest expert scores (3.04 ± 0.86) and GPT the lowest (2.30 ± 0.61). Post-hoc analyses confirmed that GPT was significantly worse than all other models in ground truth consistency (all p < 0.001, Cohen’s d = 0.95–1.72, large effect sizes), while DeepSeek-V3, GLM, and Qwen showed comparable performance without significant pairwise differences in Figure 6.

The inter-rater reliability between the two experts was assessed using Pearson’s correlation coefficient (r) and Cohen’s kappa (κ) for categorical agreement. For Task 1, substantial to almost perfect agreement was observed across all models (r = 0.828–1.000, κ = 0.536–1.000). For Task 2, GPT, Kimi, and Qwen showed perfect inter-rater agreement (r = 1.000, κ = 1.000), while DeepSeek (r = 0.705, κ = 0.553) and GLM (r = 0.713, κ = 0.209) exhibited moderate to substantial agreement, indicating greater variability in expert judgment for these models.

## 5 Discussion

The reported F1-scores of 44.89% (entity extraction) and 32.13% (formula prediction) represent moderate performance in absolute terms. These metrics should be contextualized within the inherent complexity of TCM clinical reasoning: (1) TCM syndrome differentiation involves holistic, multi-dimensional pattern recognition that resists reduction to simple entity matching; (2) formula prediction requires not only correct identification of herbal components but also precise dosage calibration and combination logic; (3) the BERTScore of 81.07% achieved by DeepSeek-V3 in semantic similarity assessment indicates strong alignment with expert reasoning patterns, which may be more clinically meaningful than strict entity matching. While our CoT-RAG framework significantly outperforms baseline models (e.g., DeepSeek-V3 without RAG achieved only 28.44% on entity extraction), there remains substantial room for improvement in absolute performance.

To capture the deep semantic nuances of TCM texts, which often use diverse terminology to describe identical syndromes, we employed BERTScore. Unlike traditional overlap-based metrics like BLEU, BERTScore evaluates the contextual similarity between generated outputs and gold standards, providing a more robust measure of clinical consistency (Zhang et al., 2019). Our RAG-driven framework provides a ‘factual anchor’ by grounding outputs in a verified TCM knowledge base (Thirunavukarasu et al., 2023). Unlike traditional black-box generative models, our approach does not merely retrieve information; it structures the generative process into a ‘Chain-of-Thought’ (CoT) that mimics the dialectical reasoning of expert physicians (Zhang et al., 2023). By leveraging retrieval-augmentation instead of static parameter updates, we effectively bypass the ‘knowledge lag’ inherent in pre-trained models and ensure source traceability. This paradigm shift, from treating LLMs as opaque oracles to designing them as knowledge-grounded clinical assistants, offers a robust and scalable solution for the responsible deployment of AI in complex medical decision-making.

Despite the significant improvements brought by RAG in our framework, completely eliminating factual inaccuracies remains a persistent challenge in medical LLMs (Huang et al., 2025). In traditional Chinese medicine, whereinteractions can profoundly affect patient safety, unregulated AI outputs pose severe risks. Therefore, aligning with the consensus of regulatory oversight (Meskó and Topol, 2023; Denecke et al., 2024), we emphasize that our TCM-CoT-RAG system is designed as a Clinical Decision Support System (CDSS) rather than an autonomous agent. A rigorous ‘human-in-the-loop’ mechanism (Olawade et al., 2026), requiring final review by licensed TCM practitioners, is indispensable for clinical deployment.

While Large Language Models (LLMs) have demonstrated remarkable potential in encoding clinical knowledge (Singhal et al., 2023), their propensity for ‘hallucinations’ remains a significant barrier to clinical adoption. Our proposed hybrid RAG framework directly addresses this by grounding the generative process in a verified TCM knowledge base, effectively acting as a ‘factual anchor’ to ensure clinical reliability. Unlike traditional single-pass information extraction, our study incorporates a Cross-Model Consistency Chain of Thought (CMC-CoT) framework (Wei et al., 2022). We treat each of the five LLMs (DeepSeek, Qwen, etc.) as an independent reasoning agent. TCM diagnosis is a complex cognitive process involving multi-step dialectical reasoning. Unlike black-box models, our approach incorporates ‘Chain-of-Thought’ (CoT) inspired components, such as dialectical analysis and mentor comments, which align with the expert reasoning patterns emphasized in contemporary TCM AI research (Han et al., 2026). This explainability is crucial for clinical trust. Our results demonstrate that a hybrid strategy, combining retrieval augmentation with domain-specific fine-tuning, outperforms single-modality approaches. This synergy allows the model to leverage external factual knowledge while maintaining the specialized linguistic and logic nuances of TCM practitioners learned (Lewis et al., 2020). Beyond clinical assistance, our framework represents a digital paradigm for preserving and analyzing expert physicians’ experiences. By integrating RAG with expert logic, we facilitate a more structured exploration of, aligning with the principles of network pharmacology and AI-driven pharmaceutical innovation (Hopkins, 2008; Lu et al., 2023).

## 6 Limitations

The current system is bounded by the specialized records of a single renowned expert in rheumatology. While this ensures high logical fidelity within a specific domain, it may limit generalizability to broader TCM sub-disciplines. Future iterations must incorporate multi-center, multi-expert datasets to mitigate potential domain bias and enhance algorithmic robustness (Topol, 2019). While BERTScore is useful for measuring semantic fidelity, it cannot fully evaluate clinical correctness or patient safety. Additional evaluation by domain experts, prescription appropriateness assessment, or inter-rater agreement analysis is essential to strengthen the clinical validity of the proposed framework.

Our evaluation relied primarily on internal expert assessment and automated metrics. External validation using independent datasets is planned and preliminary results will be reported in Supplementary Appendix 4. Additional evaluation by domain experts, prescription appropriateness assessment, or inter-rater agreement analysis is essential to strengthen the clinical validity of the proposed framework. Furthermore, the expert human validation (Section 4.4) provides complementary assessment of clinical quality that partially addresses these metric limitations, though subjective expert evaluation introduces its own variability and potential bias. Combining quantitative automatic metrics and expert qualitative evaluation results: The external validation achieved Entity F1 = 38.5% (difference = −6.4%), Formula F1 = 26.2% (difference = −5.9%), BERTScore = 76.3% (difference = −4.8%). All quantitative performance declines are controlled within 10%; meanwhile, all expert evaluation scores exceed 3.5 points, and inter-rater reliability reaches an acceptable level. Collectively, the external validation shows acceptable generalization with slight performance degradation of 5.7% (average differential of three metrics), which supports the framework’s applicability to external clinical settings and published expert medical record scenarios.

Although the RAG architecture mitigates knowledge lag, our current retrieval base is not continuously updated. Developing dynamic, real-time ingestion mechanisms for evolving clinical guidelines is essential for real-world deployment. TCM diagnosis relies heavily on visual (e.g., tongue inspection) and auditory cues. Transitioning from text-only inputs to multimodal medical foundation models capable of synergizing image, voice, and text data represents a significant frontier for developing a fully integrated TCM assistant (Moor et al., 2023). Ultimately, this work demonstrates that the convergence of retrieval-augmented generation, structured reasoning frameworks, and human expertise offers a robust and scalable pathway toward responsible AI deployment in traditional medicine, bridging the ancient wisdom of TCM with the demands of modern healthcare digitization.

## Data Availability

The original contributions presented in the study are included in the article/Supplementary Material, further inquiries can be directed to the corresponding authors.
