# Supplementary material for: TCM-CoT-RAG: a chain-of-thought enhanced retrieval-augmented generation system for clinical decision support in Traditional Chinese Medicine rheumatology
Source: Front Pharmacol. 2026 Aug 12;17:1884526. doi: 10.3389/fphar.2026.1884526 (PMC13507730; doi:10.3389/fphar.2026.1884526)
Supplement: Supplementary file 2 [file DataSheet2.pdf]

Appendix A

Dimension	Score	Criteria
**Syndrome Differentiation Quality**	5	Fully consistent with expert judgment; logical coherence complete
	4	Largely correct with minor imperfections
	3	Partially correct; requires modification
	2	Significant errors in reasoning
	1	Completely incorrect or clinically unsafe
**Prescription Appropriateness**	5	Clinically sound; appropriate botanical drug selection and dosage
	4	Generally appropriate with minor adjustments needed
	3	Partially appropriate; some concerns about drug selection or dosage
	2	Multiple inappropriate elements
	1	Clinically inappropriate or potentially harmful
**Alignment with Ground Truth**		The "Senior Title Ground Truth Consistency" score evaluates the matching degree between the model's predicted diagnosis (Western medical disease names + TCM syndrome differentiation) and the manually annotated standard case core points (Ground Truth). Three grading standards are adopted.
**Diagnosis Module Consistency (Case Diagnosis Matching)**	2	The model fully covers all standard Western diagnoses, TCM syndromes, combined complications and medical history in the case; no misdiagnosis or missing core lesion labels.
	1	The model correctly identifies the primary core disease of the case, but partially omits secondary syndromes, concurrent diseases or historical lesions; no misjudgment of the dominant diagnosis.
	0	The model misdiagnoses or completely ignores the core disease recorded in the standard case; the predicted diagnosis deviates fundamentally from the real clinical orientation of the case.
**Prescription Module Consistency (Herb Hit Rate Matching)**		The consistency metric for prescription output is defined as herb hit rate, calculated by counting the number of overlapping core medicinal herbs between the model-generated formula and the standard therapeutic prescription corresponding to the case Ground Truth. Grading criteria based on the count of

		matched herbs.
	2	≥8 overlapping core herbs between model prescription and standard formula; herb hit rate ≥ 80%. The formula's therapeutic principle fully fits the primary and secondary pathogenesis of the patient.
	1	3–7 overlapping core herbs; herb hit rate ranges from 30% to 79%. The formula matches the therapeutic logic of the main disease, yet lacks key medicinal ingredients for concurrent symptoms and secondary syndromes.
	0	0–2 overlapping core herbs; herb hit rate ＜ 30%. The formula's therapeutic direction is inconsistent with the core pathogenesis of the standard case, with extremely few effective matching medicinal materials.

Appendix B

Syndrome Differentiation Quality

**Evaluator Pair**	**Cohen's κ**	**Interpretation**
Expert 1 – Expert 2	0.933	Almost Perfect
Expert 1 – Expert 3	0.567	Moderate
Expert 2 – Expert 3	0.551	Moderate
Fleiss' κ (overall)	0.712 (95% CI: 0.651 – 0.773)	Substantial

Prescription Appropriateness

**Evaluator Pair**	**Cohen's κ**	**Interpretation**
Expert 1 – Expert 2	0.933	Almost Perfect
Expert 1 – Expert 3	0.567	Moderate
Expert 2 – Expert 3	0.551	Moderate
Fleiss' κ (overall)	0.712 (95% CI: 0.651 – 0.773)	Substantial
