# Supplementary material for: TCM-CoT-RAG: a chain-of-thought enhanced retrieval-augmented generation system for clinical decision support in Traditional Chinese Medicine rheumatology
Source: Front Pharmacol. 2026 Aug 12;17:1884526. doi: 10.3389/fphar.2026.1884526 (PMC13507730; doi:10.3389/fphar.2026.1884526)
Supplement: Supplementary file 3 [file DataSheet3.pdf]

# RAG System Technical Parameters

## 1. Retrieval Hyperparameters

### Top-k Retrieval Parameters
| Component | Top-k Value | File Location |
|-----------|-------------|---------------|
| Reference Retrieval (RagReference) | k=3 | rag_reference.py:276 |
| Classical Book Retrieval (RagClassicalBook) | k=2 | rag_classical_book.py:671 |
| Similar Case Search | k=3 | prompt_create.py:155 |
| Classical Book Keyword Search | k=2 | prompt_create.py:220 |

### Retrieval Strategy
- **Method**: Dense vector similarity search via Chroma vector database
- **Retrieval Function**: `similarity_search_with_score(query_text, k=k)`
- **Hybrid Search**: Combines BGE-M3 dense vector retriever with BGE-Reranker (cross-encoder) for re-ranking

---

## 2. Chunking Strategy

### Text Splitting Configuration
| Parameter | Value | File Location |
|-----------|-------|---------------|
| Chunk Size | 1000 characters | rag_reference.py:165, rag_classical_book.py:521 |
| Chunk Overlap | 200 characters | rag_reference.py:166, rag_classical_book.py:522 |
| Splitter Type | RecursiveCharacterTextSplitter | langchain |

**Code Reference**:
```python
# rag_reference.py:164-168
self.text_splitter = RecursiveCharacterTextSplitter(
    chunk_size=1000,
    chunk_overlap=200,
    length_function=len
)
```

---

## 3. Embedding Configuration

### Embedding Model Specifications
| Parameter | Value | Source |
|-----------|-------|--------|

| Embedding Model | text-embedding-v4 | setting.yaml:8 |
| Embedding Dimension | 1536 | openai_api.py:174 |
| API Provider | DashScope (Alibaba Cloud) | setting.yaml:4 |
| Encoding Format | float | openai_api.py:142 |

**Code Reference**:
```python
# openai_api.py:138-143
response = client.embeddings.create(
    model="text-embedding-v4",
    input=text,
    encoding_format="float"
)
```

---

## 4. BM25 Configuration

**Note**: This system uses **pure dense vector retrieval** via Chroma, NOT BM25. The hybrid search mentioned in the paper refers to the combination of:
- Dense vector retrieval (BGE-M3)
- Cross-encoder reranking (BGE-Reranker)

There is **no BM25 keyword-based retrieval** in the current implementation.

---

## 5. Model Hyperparameters

### LLM Configuration
| Parameter | Value | File Location |
|-----------|-------|---------------|
| max_tokens | 8000 | setting.yaml:16 |
| temperature | 0.01 | desease_main.py:89 |
| seed | 42 | setting.yaml:21 |

### Models Used
| Model | Version/Config | API Endpoint |
|-------|---------------|---------------|
| Qwen (Moonshot-Kimi-K2-Instruct) | Moonshot-Kimi-K2-Instruct | DashScope |
| DeepSeek | DeepSeek-V3 | DashScope |
| GLM | glm-4-flash | BigModel.cn |
| GPT | Not used in code | - |

## 6. Fine-tuning Process

**Status**: NOT APPLICABLE

This system does **NOT** perform any fine-tuning. All models are accessed via API calls:
- Qwen (Moonshot-Kimi-K2-Instruct) via DashScope API
- DeepSeek-V3 via DashScope API
- GLM-4-flash via BigModel.cn API

The system relies on:
1. **Prompt engineering** with chain-of-thought style templates
2. **RAG retrieval augmentation** for domain-specific knowledge grounding
3. **Human-in-the-loop validation** for clinical safety

No gradient-based training or parameter updates are performed.

---

## 7. Computational Resource Specifications

| Resource | Specification | Notes |
|----------|---------------|-------|
| Deployment Platform | Alibaba Cloud| DashScope API |
| Database | Chroma Vector Database | Local persistence |
| Cache | MySQL | For embedding caching (optional) |
| MySQL Server | 192.168.28.2:3333 | llm_cache database |
| API Rate Limit | 60 requests/min | setting.yaml:15 |
