# Supplementary material for: TCM-CoT-RAG: a chain-of-thought enhanced retrieval-augmented generation system for clinical decision support in Traditional Chinese Medicine rheumatology
Source: Front Pharmacol. 2026 Aug 12;17:1884526. doi: 10.3389/fphar.2026.1884526 (PMC13507730; doi:10.3389/fphar.2026.1884526)
Supplement: Supplementary file 4 [file DataSheet4.pdf]

Appendix 4. External Validation Protocol and Evaluation Methods

4.1 Rationale for External Validation

To demonstrate the generalizability of the TCM-CoT-RAG framework beyond the internal evaluation dataset, external validation is essential. The internal validation results, while encouraging, are derived from cases collected from a single expert practitioner. External validation using independent data sources will provide evidence for the framework's robustness across different clinical settings, practitioners, and documentation styles.

4.2 External Validation Dataset Requirements

The external validation dataset should meet the following criteria:

1. **Data Source:** 50 published expert rheumatism medical records from Peking University Core journals, distinct from Professor Fang's clinical records used in the internal evaluation.
2. **Sample Size:** A sample of 50 published expert rheumatism medical records from Peking University Core journals, ensuring each case includes complete four diagnostic information, syndrome differentiation, and botanical drug prescription.
3. **Disease Coverage:** Rheumatoid arthritis, gout, ankylosing spondylitis, and other rheumatologic conditions consistent with the internal evaluation dataset.
4. **Data Format:** Each case must include: symptom description, tongue and pulse findings, TCM syndrome differentiation (ground truth), and botanical drug prescription (ground truth).

4.3 Evaluation Metrics and Calculation Methods

4.3.1 Entity Extraction F1 Score

The Entity F1 score evaluates the accuracy of TCM syndrome differentiation and key clinical entity extraction. Given the model-predicted entities (syndromes, symptoms, treatment principles) and reference entities (ground truth), F1 is calculated as:

$$Precision = TP / (TP + FP),\ Recall = TP / (TP + FN),\ F1 = 2 \times (Precision \times Recall) / (Precision + Recall)$$

Where TP = true positives (correctly matched entities), FP = false positives (incorrectly predicted entities), and FN = false negatives (missed entities). For TCM syndrome entities, exact string matching is used for primary syndromes, while partial matching is applied to modifier terms (e.g., "Cold" in "Cold-Damp").

4.3.2 Formula Prediction F1 Score

The Formula F1 score evaluates prescription similarity at the individual botanical drug level. Given the predicted prescription (list of herbs) and reference prescription (ground truth), drug-level F1 is calculated:

$$Precision = |Predicted\ Drugs \cap Reference\ Drugs| / |Predicted\ Drugs|$$

$$Recall = |Predicted\ Drugs \cap Reference\ Drugs| / |Reference\ Drugs|$$

Note that dosage matching is not required for F1 calculation; only the presence of correct botanical drugs in the prescription is considered. This approach accounts for the fact that TCM prescriptions often admit multiple valid herb combinations for the same syndrome pattern.

4.3.3 BERTScore for Semantic Fidelity

BERTScore evaluates the semantic similarity between model-generated reasoning (diagnostic reasoning, experience summary, supervisor evaluation) and reference texts. Unlike exact-match metrics, BERTScore leverages contextual embeddings to capture semantic equivalence even when surface expressions differ:

Where precision and recall are computed using cosine similarity between BERT embeddings of the candidate and reference sentences. BERTScore values range from 0 to 1, with higher values indicating greater semantic similarity. For TCM clinical reasoning, a BERTScore above 0.70 indicates acceptable semantic fidelity, while values above 0.80 indicate strong alignment with expert reasoning patterns.

4.4 Expert Qualitative Assessment Protocol

Beyond automated metrics, expert qualitative assessment is essential to evaluate clinical correctness and safety. Three senior TCM rheumatologists (each with >15 years of clinical experience), who were not involved in the internal evaluation, will independently assess a random sample of model outputs using a 5-point Likert scale:

1. **Diagnostic Accuracy (1-5):** Alignment of model-generated syndrome

differentiation with TCM theory and clinical evidence.

2. **Prescription Appropriateness (1-5):** Clinical validity and safety of the recommended botanical drug prescription.
3. **Overall Clinical Utility (1-5):** Suitability of the model output as a clinical decision-support reference.

Inter-rater reliability will be assessed using Cohen's kappa (κ), with $\kappa > 0.70$ indicating acceptable agreement among raters.

4.5 Expected Outcomes and Interpretation

Based on the internal validation results (Entity F1: 44.89%, Formula F1: 32.13%, BERTScore: 81.07%), we anticipate that external validation will show a performance differential of 5-15% due to differences in clinical documentation styles and expert reasoning patterns across institutions. The external validation results will be interpreted as follows:

Acceptable Generalizability: External F1 within 10% of internal F1; expert ratings $\geq$ 3.5/5.

Limited Generalizability: External F1 10-20% below internal F1; expert ratings 2.5-3.5/5. Indicates need for domain adaptation.

Poor Generalizability: External F1 > 20% below internal F1; expert ratings < 2.5/5. Suggests significant domain shift requiring retraining or multi-domain adaptation.

External Validation Results

Quantitative Evaluation

Metric	Internal	External	Difference
**Entity F1**	44.89%	38.5%	-6.4%
**Formula F1**	32.13%	26.2%	-5.9%
**BERTScore**	81.07%	76.3%	-4.8%

A 50 external validation records for expert blind evaluation (50% sampling rate, consistent with mainstream medical AI evaluation standards). The average scores of three evaluation dimensions are summarized in the table below, retaining two decimal places for academic rigor:

Evaluation Dimension (5-point Likert Scale)	Average Expert Score	Score Standard Deviation
**Diagnostic Accuracy**	3.72	0.31
**Prescription Appropriateness**	3.65	0.35
**Overall Clinical Utility**	3.81	0.29

All three dimension scores are higher than the threshold of 3.5/5 defined in Section 4.5, which meets the pre-specified acceptable generalizability standard.

Cohen's Kappa coefficient was calculated to quantify consistency among three independent rheumatology experts: The overall inter-rater κ = 0.74. Specifically, κ = 0.75 for diagnostic accuracy dimension, κ = 0.71 for prescription appropriateness dimension, κ = 0.76 for overall clinical utility dimension. All κ values exceed the 0.70 threshold, demonstrating substantial evaluator agreement and eliminating subjective evaluation bias caused by single-expert experience heterogeneity.

The TCM four-diagnosis entity extraction logic fits classical TCM rheumatism diagnosis specifications; the syndrome differentiation reasoning process is traceable, and most herbal prescriptions conform to monarch-minister-assistant-guide prescription principles. The CoT reasoning module effectively avoids rigid mechanical matching of medical records. For complex comorbid rheumatism cases (gout combined with rheumatoid arthritis), the model tends to ignore secondary mild cold-dampness syndrome; individual herb selections lack flexibility consistent with real-world clinical medication habits, which is the core cause of slightly decreased Formula F1 score in external validation.
